# More about Nice

**Published:** 1888-03-24

**Authors:** E. A. Woodcock

**Affiliations:** (Directress). Hollond Nursing Institution, 4, Rue Adelaide, Nice


### MORE ABOUT NICE.

As I have had many applications from nurses in consequence of the information kindly given about this institution by a correspondent in your number of March 3rd, it may be well if I correct one or two little mistakes made, and specify some of the qualifications desirable in nurses here, and so prevent disappointment to numbers who apply without being at all suitably qualified, and also spare myself much correspondence. First—the address is altered from Avenue Delphine to the one given below. Then, nurses are engaged for intervals between October 1st and June 1st, varying from eight to three months, according to agreements in each case. Applications to enter during the next season should be made early in the previous May, by letter to the Directress, at the Nice address, enclosing certificates of training and testimonials of later work, copied on thin paper, as the postage is 2½*d.* for each half-ounce. All particulars should be added as to health, age, height, weight, etc., and the address given of a referee in a responsible position. An interview with the Directress in London about the end of June is necessary before any decision can be made. It should be added that almost all the nurses engaged are daughters of clergymen, medical or other professional men; therefore, a considerable amount of general education is to be expected of them, including ability to speak French. Some knowledge also of German or Italian is desirable. As to special education in their profession, applicants should have had three years' training in a good general hospital, and private nursing in addition. A good knowledge of sick cookery is most important. The salary given is at the rate of £36 a-year, with three francs a-week for laundress; outdoor and indoor uniforms are provided, and all travelling expenses, as stated by your correspondent.

E. A. Woodcock (Directress).

Hollond Nursing Institution,
4, Rue Adelaide, Nice,

